# Feasibility, acceptability and adaption of dignity therapy: a mixed methods study achieving 360° feedback

**DOI:** 10.1186/s12904-018-0326-0

**Published:** 2018-05-10

**Authors:** Sandra Stephanie Mai, Swantje Goebel, Elisabeth Jentschke, Birgitt van Oorschot, Karl-Heinz Renner, Martin Weber

**Affiliations:** 1 Interdisciplinary Palliative Care Unit, III. Department of Medicine, University Medical Center of the Johannes Gutenberg University of Mainz, Mainz, Germany; 2 Interdisciplinary Center for Palliative Medicine, University Hospital Würzburg, Würzburg, Germany; 3 Department of Psychology, Bundeswehr University Munich, Neubiberg, Germany

**Keywords:** Dignity therapy, Palliative care, Qualitative research, Translation

## Abstract

**Background:**

Dignity Therapy (DT) is a short-term intervention to reduce psychological suffering in end-of-life care. Its strength lies in evidenced-based development and investigation. The aim of the present study is to investigate the feasibility of DT at German palliative care units (PCU), as well as the acceptability and adaption of a German version of the DT question protocol (DTQP).

**Method:**

A clinical multicentre mixed methods study, whereby patients and relatives provided quantitative (feedback questionnaires) and qualitative (cognitive interviews) data on the DT intervention. Before using the DTQP on patients, healthcare professionals (HCP) were invited to participate in cognitive interviews to provide input on DT. Therefore 360° feedback was achieved. Finally, the conducted DT interviews were examined.

The study took place at two German PCUs (Mainz and Würzburg). Participating HCPs were physicians, psychologists, nurses and chaplains. Patients admitted to the PCUs were eligible to participate if they had a terminal illness and a life expectancy ranging from 2 weeks to 12 months.

Results: Out of 410 admitted patients, 72 were eligible and 30 (7.3% of all patients and 41.7% of eligible patients) participated. On average, 9 questions from the DTQP were used per DT interview. Subsequent cognitive interviews with patients produced four main categories of feedback (on the title, the question protocol, wording, and the questions actually asked). Finally, of the 30 participants, 19 completed the feedback questionnaire, as did 26 relatives. Of those, 18 patients and 24 relatives evaluated DT as helpful.

**Conclusions:**

DT is feasible for German PCUs. Our research yielded a validated German translation of the DTQP following EORTC guidelines and findings were reported according to the COREQ checklist for qualitative design.

Trial registration.

The study was registered retrospectively on the 22nd of December 2017 at the German Clinical Trials Register (DRKS00013627).

**Electronic supplementary material:**

The online version of this article (10.1186/s12904-018-0326-0) contains supplementary material, which is available to authorized users.

## Background

Depression, anxiety, hopelessness and loss of meaning and purpose are central symptoms of the demoralization and psychological distress faced by patients in palliative care [1]. These burdens are associated with loss of dignity [2, 3]. In a systematic review, Monforte-Royo concludes that the loss of dignity and its influence on hopelessness and loss of meaning may result in a wish for hastened death [4]. An updated systematic review added that “loss of self” and the “lived experience of a timeline towards dying and death” were common features of a patient’s wish to hasten death [5]. These findings indicate that patients need appropriate supportive care to address psychological symptoms and ensure quality of life in their final days. An evidence-based intervention to care for the terminally ill on a psychosocial and spiritual basis is Dignity Therapy (DT) [6]. DT was first described in 2005 by Harvey Max Chochinov, based on his findings on dignity in the terminally ill and the dignity model [3]. The dignity model provides three major dignity categories (illness-related concerns, dignity-conserving repertoire, social dignity inventory) and several themes (e.g., dignity-conserving perspectives and dignity-conserving practices) evoked from patients’ explanations of their understanding of dignity. The basis of DT is a semi-structured question protocol (Table 1).

**Table 1:** The Dignity Therapy Question Protocol

(1)* Tell me a little about your life history; particularly the parts that you either remember most or think are the most important. (2) When did you feel most alive?	
(3) Are there specific things that you would want your family to know about you, (4) and are there particular things you would want them to remember?	
(5) What are the most important roles you have played in life (family roles, vocational roles, community-service roles, etc.)? (6) Why were they important to you, and what do you think you accomplished in those roles?	
(7) What are your most important accomplishments, and what do you feel most proud of?	
(8) Are there particular things that you feel still need to be said to your loved ones (9) or things that you would want to take the time to say once again?	
(10) What are your hopes and dreams for your loved ones?	
(11) What have you learned about life that you would want to pass along to others? (12) What advice or words of guidance would you wish to pass along to your (son, daughter, husband, wife, parents, other[s])?	
(13) Are there words or perhaps even instructions that you would like to offer your family to help prepare them for the future?	
(14) In creating this permanent record, are there other things that you would like included?	

*Numbering is only to ensure traceability of methodology and results for the reader

During an audio-recorded interview session the therapist guides the patient through these questions to enhance the dignity-conserving perspectives. Most importantly the framework of questions is intended to be flexible with a varying number of questions actually used in order to suit the individual patient ([7], p. 185). The transcript of the interview session is edited and read out to the patient; if necessary, further editing can be performed. The transcript is then finalized to create a permanent generativity document which can be shared with loved ones [7]. Research on the feasibility and efficacy of DT has been published for six countries [8]. Although findings regarding its efficacy have been contradictory in recent randomized controlled trials [8, 9], helpfulness and meaningfulness are proven benefits for patients and relatives, and DT is regarded as feasible in different clinical settings, healthcare systems and cultures. These findings were an encouraging starting point to evaluate the implementation of DT in palliative care units (PCU) in Germany.

Although DT has already been used in Germany to a limited extent, the Dignity Therapy Question Protocol (DTQP) to date lacks a scientifically verified translation into German. Various unproven translations of the question framework have been used up to this point. What is more, the feasibility and acceptability of DT in Germany have not been examined before. And yet these issues are fundamental to enable further comparable research and the implementation of DT in Germany. The purpose of this research was therefore to conduct a mixed methods study at two German PCUs, in Mainz (M) and Würzburg (W). Similar to the implementation of DT in Denmark [10], the aims of our study were (1) to translate the DTQP and if necessary adapt the question framework, and (2) to test the feasibility and acceptability of DT in an inpatient setting.

## Methods

The clinical multicentre study comprises quantitative and qualitative research at two PCUs in the university hospitals of M and W. After their actual DT interview, patients participated in semi-structured cognitive interviews to report their experience of the process through free narratives. Narratives are the most appropriate form of research to report experiences. Patients also completed a feedback questionnaire (quantitative and free text comments) to gather further information on their estimation of DT. Once the patient DT process was complete, relatives were also asked to evaluate DT by completing a feedback questionnaire similar to that of the patients. To achieve 360° feedback, healthcare professionals (HCP) from different disciplines within palliative care were interviewed in expert focus groups. This method allowed critical discussion of the different aspects arising from DT. The information detailed below follows this timeline and complies with the consolidated criteria for reporting qualitative research (COREQ) [11].

### Translation of the dignity therapy question protocol (DTQP)

In accordance with the EORTC Translation Procedure [12], two English and two German native speakers conducted forward-backward translations of the DTQP. These translations were discussed at round tables by physicians and psychologists experienced in palliative care and, where necessary, checked back with H.M. Chochinov to obtain a pilot version.

### HCP evaluation of the DTQP German translation

In June and July 2015, two experienced researchers (S.S.M., qualified psychologist, and PhD S.G., sociologist) conducted focus groups with HCPs in meeting rooms at the university hospitals of M and W; the meetings lasted 60 and 70 min respectively. Both researchers were clinical and scientific colleagues of the participants from M; however, prior to study commencement, they were not acquainted with the HCP participants from W. Participating HCPs were all highly experienced in palliative care and were purposively recruited by e-mail. The number of participants represents the multi-professionalism of palliative care teams, consisting of five HCPs in M (psychologist, chaplain, nurse and two physicians) and seven HCPs in W (two chaplains, two nurses and three physicians). All participants were informed of the aim to achieve a German translation of the DTQP and they all gave written informed consent to participate. Each participant was attributed an ID to anonymize the qualitative data. As none of the 12 HCPs had previously conducted DT, their statements can be regarded as unbiased. Following a comprehensive interview guideline (developed by S.G. according to Willis’ recommendations on cognitive interviewing [13], Additional file 1), the HCPs were requested to express spontaneous associations regarding the title of the intervention, the wording of the DT questions, and the adaption of DT to the German cultural context. As well as visually summarizing and recording the participants’ statements on flip charts during the sessions, audio recordings were also made. S.S.M. and S.G. then transcribed and analysed the qualitative data based on the principles of qualitative content analysis as described by Kuckartz [14]. This method enables the elaboration of core messages from the interview transcriptions, by using inductive and deductive approaches in a systematic manner. Only parts of the interviews pertaining to the research questions have been considered, numbered, and arranged within an Excel sheet. Analysis units from the first interview transcript were paraphrased and generalized, and redundancies were identified, and inductively subsumed into main categories and subcategories. This resulting code structure was used to analyse the second interview deductively, and new codes were added inductively to the code structure, as needed. Typical phrases were identified. In the first instance both researchers coded independently. To achieve interrater reliability, categories were discussed and any coding conflicts were resolved. This process was repeated until the final structure of categories was formed and all relevant parts of the text were coded appropriately. The research group reviewed the results to ensure inter-subjectivity and coherence within the code structure. The results constituted the first step in the linguistic and cultural adaption of the DTQP.

### Patient evaluation of the DT intervention and the DTQP

Leaning on the experiences and data of the Danish DT feasibility study [10], we decided to include 30 patients from two PCUs in our study. Each patient admitted to the PCUs in M and W was attributed an ID and was screened for eligibility. Inclusion criteria were: having a terminal illness with a clinically estimated life expectancy of up to 12 months; age ≥ 18 years; ability to give informed consent; possibility to nominate a relative for the feedback questionnaire; and appropriate German language skills. Exclusion criteria were: having cognitive impairments or an estimated life expectancy below 2 weeks, especially where the dying process had begun. After giving written informed consent, patients participated in the audio-recorded DT interview led by DT therapists (M: psychologist and male nurse; W: psychologist) trained by H.M. Chochinov. All steps of the DT protocol were fulfilled (interview session, transcribing, editing, reading out to the patients, finalizing the document and taking it to the recipient). Detailed information about the duration of the DT interview and DT intervention was logged. Furthermore each question asked during the DT interview was counted, repetitions were listed, and rewording as well as patients’ noteworthy reactions were noted.

After the DT interview using the DTQP, the participants took part in semi-structured cognitive interviews with their therapists to report their experience of the implementation of the DT interview (the interview guideline was produced by S.S.M. and S.G. according to Willis’ recommendations on cognitive interviewing [13], Additional file 2). All sessions took place in the patients’ single rooms at the PCUs. The cognitive interviews were recorded, transcribed and analysed by S.S.M. and S.G. using qualitative content analysis [14] as described above. The basic coding tree results from the major themes of the cognitive interview. The resulting data constituted the second step in the adaption of the DTQP.

### Patient and relative feedback questionnaires

After delivery of the finalized generativity document, patients and relatives (as nominated by the patients and after having given informed consent) were asked to complete the DT Patient Feedback Questionnaire [15] and the DT Family Feedback Questionnaire [16] respectively. Both questionnaires previously had been translated into German by forward-backward translation (Additional file 3 and Additional file 4). They combine quantitative items (5-point Likert scale; 1 = strongly disagree, 5 = strongly agree) and free text comments.

All data were analysed descriptively with SPSS 22.0 and Microsoft Excel®.

## Results

The following results are presented in order of the described methodology and timeline.

### HCP views on the DTQP

Besides general remarks on the interpretation of the word ‘dignity’, which some HCPs viewed in a religious way and others in a more philosophical way, we observed five main categories of comments on DT and the DTQP: (1) negative aspects of DT; (2) positive aspects of DT; (3) Dignity Therapist; (4) conducting DT; and (5) the DTQP (Table 2). There were no comments on financing DT. Among the focus group conclusions was the opinion that the PCU is an appropriate setting for DT, as the patients have single rooms. HCPs also agreed that DT could be an essential part of the interdisciplinary care for patients if it is conducted with compassion: “If DT is conducted with heart, it could be an essential part of care and it becomes effective through this [compassion] and may be guided less by the questions that are asked but by the issue that lies on the patient’s heart” (M5). One HCP compared DT to the concept of ‘holding spaces’: “You give space for life issues, experiences and suffering, and this is dignifying and a valuable encounter” (W1).

**Table 2:** Themes, frequencies and example quotations of HCP views on the *DTQP (*M1/W1 = chaplain; M4 = psychologist; M3/M5/W0/W3/W6 = physician; M2/W5 = nurse)

Main Category	Themes	Frequency M / W	Example Quotation (ID)
Negative aspects of DT	Complex for patients and therapists	2 / 1	The time-consuming process is a disadvantage. (M4); Do we need such a sophisticated concept or don’t you hit on it yourself when getting a serious diagnosis? (W0)
	Psychological burden could arise	3 / 5	Negative memories could arise and create psychological burden. (M3); DT could have side effects if unconscious aspects arise. (W6)
	Patients fear negative consequences if they decline DT	3 / -	Some patients tell me they’re afraid of being treated less well if they decline something, e.g. students’ teaching courses. (M1)
	The name of the intervention is inappropriate	2 / 5	You can’t say DT. This seems inappropriate. (M1); Therapy [in the name of the intervention] is something that people don’t want to have, because they had enough therapy during their illness. (W5)
	Application of DT is limited	- / 5	DT is only appropriate for patients with the ability to communicate verbally, be self-reflective and discuss value-based issues. (W3)
Positive aspects of DT	DT encourages self-reflection	6 / 4	It triggers self-reflection, which is an advantage. (M5); DT is a process to realize what is important in my life, what is personally valuable for me. (W1)
	Generating a legacy	3 / -	The form (written words) creates the possibility to pass something on to your relatives that you couldn’t verbalize. (M2)
	DT creates space for a dignifying encounter	3 / 4	DT is about caring for the person. (M1); The concept of the dignifying attitude we find in DT is a good thing. (W3)
Dignity Therapist	Challenges for the therapist posed by DT	4 / 6	The therapist must be very sensitive to decide which statement is meant for the document. (M5); You need to know how to handle negative issues when uncovering negative affect. (W0)
	Consequences for the therapist after DT	2 / -	The interviewer may take on some of the patient’s distress. (M4)
Conducting DT	Application site / setting	1 / 5	The thousands of people in nursing homes or wards other than palliative care units should also be able to receive DT. (M1); The questions are great. I even used them during a dialogue about anamnesis. (W6)
DT Question Protocol	(Question) phrasing	11 / 8	Some questions sound awkward. (M1); Subjunctive phrases are irritating. (W2)
	Open-ended questions are stimulating	6 / 3	The first question is a good opening as it is an open-ended question. (M3); For me, open-ended questions are important … they can be heart-opening. (W1)
	Focus on generating legacy	1 / 2	The advantage is to receive a treasure of life experience, e.g., question 11. (M3); [As a participant] I’d wish to know that it doesn’t have to be a permanent record for the next generation. (W6)
	Focus on accomplishments	2 / 6	Asking for accomplishments and roles is risky when interviewing a patient who is depressed. (M4); To name something as an accomplishment as an observer from the outside, that is social dignity. (W4)
	Application by DT Therapists	6 / 6	I understood that the therapist uses some but not all of the DT questions. (M1); The effect of the questions depends on asking these questions with a warm, calm and empathic tone. (W6)

### Participant characteristics and DT intervention data

A total of 410 patients were admitted to the PCUs in M (215 patients between June 2015 and October 2015) and W (195 patients between June 2015 and April 2016). Of these, 72 patients (17%) (M: 54; W: 18) met the inclusion criteria, and 30 of them (M: 19; W: 11) participated (7.3% of all patients and 41.7% of eligible patients). Participants’ inpatient stay ranged from 5 days to 98 days (median = 16 days). The mean age was 63 years (range: 38–88); 20 participants were female; the median Eastern Cooperative Oncology Group performance status (ECOG) was 3 with a 40% Palliative Performance Scale (PPS) rating. Table 3 provides further information on the participants’ characteristics.

**Table 3:** Study participant (patients and relatives) characteristics

Patients (*N* = 30)				
Age in years		Mean: 63	SD: 9.9	Range: 38–88
Gender	Female: 20 (67%)		Male: 10 (33%)	
ECOG at admission	Median: 3	Mean: 2.77	SD: 0.73	Range: 1–4
PPS at admission	Median: 40	Mean: 45	SD: 12.8	Range: 30–80
Period of time in months *(years)* between first diagnosis and first DT interview		Mean: 33 *(2.76)*	SD: 39.29 *(3.28)*	Range: 1–163 *(0.06–13.62)*
Main diagnosis	Gastrointestinal cancers:		10 (33.3%)	
	Gynaecological cancers:		8 (26.7%)	
	Lung cancers:		5 (16.7%)	
	Urology cancers:		4 (13.3%)	
	Neurological diseases		2 (6.7%)	
	Dermatological cancers:		1 (3.3%)	
Length of inpatient stay in days	Median: 15.5	Mean: 22.5	SD: 18.0	Range: 5–98
Relatives (N = 30)				
Age in years (2 missings)		Mean: 54	SD: 12.83	Range: 24–74
Gender	Female: 14 (46.7%)		Male: 16 (53.3%)	
Relationship to patient	Spouse:		17 (56.7%)	
	Child:		6 (20%)	
	Sibling:		3 (10%)	
	Parent:		2 (6.7%)	
	Other:		2 (6.7%)	
Duration of relationship in years (5 missings))		Mean: 35.48	SD: 17.31	Range: 8–71

The main exclusion criteria were: a life expectancy below two weeks (196 patients; 58%), followed by cognitive impairments (74 patients; 22%). A further 10% of eligible patients could not be included due to organizational barriers.

The median time-span of the whole DT process – from introducing it to the patient to returning the generativity document – was 15 days (mean: 24; range: 6–131). During this time, there were four contact sessions between patient and therapist on average. The mean time expenditure for therapists was 7 h (SD: 1.6; range: 4.27–10.38), comprising time to interview, edit, read out to the patient, make final editorial changes and return the finalized document to the recipient (note that this excludes time taken to transcribe the audio recording; this task was conducted by student assistants). The mean time expenditure for the patients was 2.15 h (SD: 0.5; range: 1.25–3.5). The mean time required for the DT interview was 48 min (SD: 19; range: 20–81). Once finalized, 19 of the generativity documents were given to the patients solely (to be handed out to the relatives by themselves or after their death), 3 documents were given to patients and relatives together, and 8 were received directly by relatives.

On average, nine questions were used per interview. Q1 (“Tell me a little about your life history; particularly the parts that you either remember most or think are the most important”) was always asked; the least asked question was Q6 (“Why were [these roles] so important to you and what do you think you accomplished within those roles?”). Q2 (“When did you feel most alive?”) was asked in 24 out of 30 interviews. In one interview, to match the language used by the patient, the wording changed from “alive” to “bloody good” (M 129). In 17 out of 23 interviews, Q3 (“Are there specific things that you would want your family to know about you?”) was responded to with comments like, “I don’t have any secrets”, “I think they know everything”, or “I don’t hide anything”. Q7 (“What are your most important accomplishments, and what do you feel most proud of?”) was used 26 times. We found that Q8 (“Are there particular things that you feel still need to be said to your loved ones?”) was sometimes personalized, e.g. “to your son” (5/16). Q10 (“What are your hopes and dreams for your loved ones?”) was used 27 times and reworded 17 times (from “dreams” to “wishes”). Similarly, Q12 (“What advice or words of guidance would you wish to pass along to your …?”) was used in 11 interviews and reworded (skipping ‘words of guidance’) without prior agreement between the interviewers.

### Patient views on the DTQP and the DT intervention

Out of 30 patients, 29 subsequently took part in cognitive interviews to share their views on the DTQP (mean duration: 10 min; SD: 6; range: 2–30). We identified four main categories of feedback: on the title; the DTQP; the wording of individual questions; and the set of questions actually asked during the DT interview (Table 4). In addition, patients gave the following positive remarks and ideas regarding DT: the questions could lead to more openness in talking about cancer; DT preserves pride; DT should be widely offered; DT should be conducted in the presence of relatives; DT is only successful if it is a helpful encounter. More sceptical comments related to the effectiveness or usefulness of DT or to the fear of psychological challenges arising. One patient suggested adding the following question: “What has been your mission in your life?” (M 43). Regarding Q5, she recommended changing “remits” into “roles” (M 43); this was the de facto wording in 8 out of 23 interviews. For comparative purposes, Q7 (“What are your most important accomplishments, and what do you feel most proud of?”) was of particular interest: Danish colleagues found that using the word “proud” was not feasible for the Danish culture [10 Pride was only discussed as negative respectively positive by 2 patients each. Case M 136 illustrates this issue. Within the cognitive interview the patient stated that “pride is too strong” and that “it should be replaced with contentment”. Yet, during the DT interview she had easily answered that question by recounting what she had achieved on her own after getting divorced. The interview atmosphere was clearly not negatively affected by the word “proud”; in contrast it seemed to offer her the opportunity to be proud of herself.

**Table 4:** Themes, frequencies and example quotations of patient views on the DTQP (*N* = 29)

Main Category	Themes	Frequency M / W	Example Quotation (ID)
Title of the intervention (Dignity Therapy ➔ Würdezentrierte Therapie)	The title conveys dignity until the end	1 / -	The title conveys that my entire existence is dignified and I am taken seriously as long as I am alive. I want to be appreciated. (M 136)
	Dignity conveys respect	6 / 1	Dignity means to accept the person and respect him or her as he or she is. (M 163)
	Dignity is an attitude	4 / -	Dignity evokes the idea of appreciating your own life story. (M 128)
	Therapy is irritating	4 / -	Therapy is an action and I don’t see how this goes together with dignity. (M 202)
	Therapy conveys help	11 / 2	Therapy conveys a helpful technique to solve a problem. (M 43)
	The title is not informative	8 / 2	First I couldn’t understand anything with the title. I had no idea what kind of technique this therapy could be. (M 43)
	The title is suitable	8 / 5	The title is suitable for this intervention as it means to reflect about what was important in your life. (W 23)
Dignity Therapy Question Protocol	Everything is addressed	3 / 2	The questions cover everything like a comprehensive frame. (M 99)
	Open questions provoke reflection	4 / 1	The questions force you to think back and reminisce about what you have gone through. (W 54)
	The DTQP is coherent	5 / 7	I think it was coherent and every question fits; it combines theoretical and practical aspects. (M 117)
	The questions in the DTQP make sense	6 / 1	The questions are all emotional, some make you cry, some make you laugh about something, but it’s always liberating. (W 23)
	The DTQP contains duplications	3 / 2	Question no. 8 and no. 9 are similar. (W 007)
	Some questions are difficult	5 / 2	It’s difficult to respond to questions that are partly very personal – but it works. (M 83)
	There are no upsetting questions	5 / -	The questions were alright. None of them was disruptive. (M 92)
Wording of individual questions	There are no disruptive words	3 / -	There were no specific words that were upsetting. (M 21)
	Question 5: Change “Remit” (*Aufgabenbereiche*) to “Roles” (*Rollen*)	1 / -	I think it would be better to ask for roles instead of remits. (M 43)
	Question 2: The word “alive” (*lebendig*) is moving	3 / -	I like the word ‘alive’ because it encompasses everything and is different from being happy or cheerful. (M 57)
	Question 8, 9, 10: Relatives mentioned should be personalized	- / 1	For me, I would replace relatives with children. (W 007)
	Question 7: “Accomplishments” (*Leistungen*) and “Pride” (*Stolz*) are connoted positively	2 / -	Pride and accomplishments belong to us by nature; it’s something positive. (M 129)
	Question 7: “Pride” (*Stolz*) is connoted negatively	2 / -	The word “pride” is too strong and should be replaced with contentment. (M 136)
	Question 12: “Advice” (*Rat*) should be replaced	- / 1	The word ‘advice’ is disruptive because I think everyone lives his or her own life. (W 23)
Set of questions actually asked during DT interview	The photo metaphor is well received	1 /	The question regarding memories as if looking through a photo album is nice. (M 43)
	All questions elicited a response	3 / -	Any question asked was okay, so that it [the interview] was genuine. (M 83)
	Questions concerning loved ones are emotionally evocative	3 / 1	It is important to talk about the emotional things although they trigger sadness. (W 23)
	Additional questions arise situational	3 / -	The DTQP provides the questions to give me an idea of what the interview is about – additional questions come up as we go along. (M 129)
	The interview atmosphere contributes to a successful DT interview	3 / -	The Therapist was very good; I don’t think I have opened up that much with many people. (M 136)

Of the 30 patients who took part in the DT interview, 19 returned feedback questionnaires. In all, 18 patients evaluated DT as helpful (“It was helpful, because DT gave me a feeling of stability” [M 43]) or satisfactory (“It was far more than satisfactory. DT is a gift. It was more than I expected” [M 43]); 14 patients agreed that DT heightened their sense of dignity (“I feel accepted the way I am” [W 42]). All patients were satisfied with their psychosocial care. Statistics and selected comments for all items are presented in Table 5.

**Table 5:** Patient feedback questionnaires on DT

Item	*N*	strongly agreed or agreed (*n* / %)	disagreed or strongly disagreed (*n* / %)	neither agreed or disagreed (*n* / %)	Selected Comments (ID)
I have found Dignity Therapy to be helpful to me.	19	18 / 94.7	–	1 / 5.3	It was helpful, because warm memories became present; it gave me a feeling of stability. (M 43)
I have found Dignity Therapy to be satisfactory.	19	18 / 94.7	1 / 5.3	–	It was far more than satisfactory. DT is a gift. It was more than I expected. (M 43)
Dignity Therapy made me feel that my life currently is more meaningful.	19	14 / 73.7	2 / 10.5	3 / 15.8	Life had the same meaning before [DT]. (M 43)
Dignity Therapy has given me a heightened sense of purpose.	19	13 / 68.4	2 / 10.5	4 / 21.1	I see that what I did wasn’t that wrong after all. (M 47)
Dignity Therapy has given me a heightened sense of dignity.	19	14 / 73.7	3 / 15.8	2 / 10.5	I feel accepted the way I am. (W 42)
Dignity Therapy has lessened my sense of suffering.	19	11 / 57.9	3 / 15.8	5 / 15.8	DT has enhanced issues that help cope with the situation. (M 43) Cancer stays cancer and death is unstoppable. (M 83)
Dignity Therapy has increased my will to live.	19	13 / 68.4	6 / 31.6	–	My way of thinking has changed. (M 83)
I believe Dignity Therapy has or will be of help to my family.	19	14 / 73.7	2 / 10.5	3 / 15.8	I hope that my family’s cohesion is strengthened [by DT]. (M 21)
I believe my participation in Dignity Therapy could change the way my family sees or appreciates me.	19	11 / 57.9	4 / 21.1	4 / 21.1	The appreciation of my family will not be affected by the therapy. (M 128)
I believe my participation in Dignity Therapy could change the way my healthcare providers see or appreciate me.	19	8 / 42.1	4 / 21.1	7 / 36.8	I think that healthcare providers won’t base their appreciation for a patient on his/her participation in DT. (M 128)
In general, I have been satisfied with my psychosocial care.	19	19 / 100	–	–	I always received help and was never ever let down. (W 42)

### Family member feedback questionnaires on the DT intervention

Out of 30 nominated relatives, 26 provided feedback and 23 of these evaluated DT as helpful for their loved ones (“To reminisce has brought up many smiles and shown how beautiful his life was and how precious life is.” [M N99]); 20 relatives agreed that DT was as important as any other aspect of care (“To talk about former times reduced spiritual pain.” [W N23]). Further, 24 relatives would recommend DT to other patients and families (“All terminally ill should receive help in this wonderful way.” [M N128]). Statistics and selected comments for all items are presented in Table 6.

**Table 6:** Family member feedback questionnaires on DT

Item	*N*	strongly agreed or agreed (*n* / %)	disagreed or strongly disagreed (*n* / %)	neither agreed or disagreed (*n* / %)	Selected Comments (ID)
I believe Dignity Therapy was helpful to my loved family member.	26	23 / 88.5	–	3 / 11.5	To reminisce has brought up many smiles and shown how beautiful his life was and how precious life is. (M N99)
I believe Dignity Therapy helped to give my family member a heightened sense of purpose or meaning in his life.	26	16 / 61.5	–	10 / 38.5	One week after, my father changed his decision to hasten death and decided to fight again. (M N21)
I believe Dignity Therapy helped to increase my family member’s sense of dignity*.*	26	16 / 61.5	1 / 3.8	9 / 34.6	To “work” within an interview was an accomplishment for her that she made because her testimonies were important. (M N136)
I believe Dignity Therapy helped prepare my family member for death.	26	17 / 65.4	–	9 / 34.6	In doing [DT] my beloved wife could reminisce about various situations or moments while she was dealing with her own death. (M N68)
I believe Dignity Therapy was as important a component of my family member’s care as any other aspect of their care, including pain management.	26	20 / 76.9	1 / 3.8	5 / 19.2	To talk about former times reduced spiritual pain. (W N23) Intensive care and pain management make suffering and pain bearable for the patient. (M N128)
I believe Dignity Therapy helped reduce my family member’s suffering.	26	14 / 53.8	1 / 3.8	11 / 42.3	Not really reducing the suffering – but in a way bringing comfort. (M N43)
Dignity Therapy helps me during my time of grief.	25	13 / 52.0	1 / 4.0	11 / 44.0	To hold something in your hand does you some good. (W N86)
Dignity Therapy will continue to be a source of comfort for my family and me.	25	16 / 64.0	3 / 12.0	6 / 24.0	It will because it is a kind of ‘love-legacy’ of my godmother. (M N136)
I would recommend Dignity Therapy to other patients or family members who are dealing with a terminal illness.	26	24 / 92.4	1 / 3.8	1 / 3.8	All terminally ill should receive help in this wonderful way. (M N128)

### Establishment of the final consensus version

The collected data led to the establishment of a consensus version, discussed by a DT expert group (S.S.M., S.G., E.J., and Jan Gramm and Jochen Spang). Slight changes to the intermediate translated version of the DTQP were made for Q3, Q4, Q5, Q8, Q9, Q10 and Q12. As “proud” was not a sensitive issue for HCPs or patients, Q7 remained. The final German version of the DTQP is provided as Additional file 5.

## Discussion

This is the first report evaluating DT in Germany. DT is a feasible intervention for PCUs in Germany and has demonstrated high levels of acceptability among patients, relatives and HCPs. The German DTQP has now been proved ready for the implementation of DT in Germany; it also provides a starting point for further research. Our methodological procedure included independent double forward-backward translation, discussions with HCPs, cognitive interviews with patients on their experience of the DT interview and the set of questions they were asked, and finally, general feedback on DT as an intervention from both patients and their family members. By providing the cognitive interview guidelines (Additional file 1 & Additional file 2), we want to contribute to further research projects on the implementation of DT in different international linguistic and cultural settings. The aspects of acceptability (for HCPs) and helpfulness and meaningfulness (for patients and relatives) were supported within this sample, although this result is limited by a small sample, mainly of patients suffering from cancer. Overall, 30 out of 72 (41.7%) eligible patients participated. For the aspect of feasibility, our results are encouraging compared to a two-year Danish study on DT that included 80 out of 389 eligible patients (23.5%) [17], as well as to a study by Hall [18], where 45 out of 188 (24%) patients responded. However, in a Portuguese study by Julião et al. only 4 of 92 patients declined to participate [9]. As we found within the cognitive interviews, DT is an intervention that is most successful through a respectful relationship between the therapist and the patient; it has to be taken into account that the effectiveness of the intervention might be influenced by the interviewing therapist. The need for further research into the efficacy of DT from the viewpoint of different HCPs and in diverse settings is described at length by Fitchett [8]. The mechanism of action of DT on psychological and spiritual needs is also largely unexplored. Wang et al. have identified a lack of randomized controlled trials on life review interventions in general and especially for DT [19]. So far, only a Portuguese study found significant reductions in psychological distress by DT [9].

## Conclusions

In summary, DT appears as a feasible and promising intervention in Germany – as well as in numerous other countries – for patients approaching the end of life. Further research on the mechanism of action and suitable methods to elucidate its effects on patients and relatives is highly warranted. Finally, there is also a need to further evaluate sources of funding for DT, as well as the impact of an extensive implementation.

## Additional files

Additional file 1: Interview guide for HCP focus groups. (DOCX 39 kb)

Additional file 2: Interview guide for patient cognitive interviews. (DOC 80 kb)

Additional file 3: DT Patient feedback questionnaire. (DOC 99 kb)

Additional file 4: DT Family feedback questionnaire. (DOC 95 kb)

Additional file 5: German version of the Dignity Therapy Question Protocol. (DOC 24 kb)

## Abbreviations

COREQ: Consolidated criteria for reporting qualitative research
DT: Dignity Therapy
DTQP: Dignity Therapy Question Protocol
EORTC: European Organisation for Research and Treatment of Cancer
HCP: Healthcare professionals
M: Mainz
PCU: Palliative care unit
W: Würzburg
