# Supplementary material for: Feasibility, acceptability and adaption of dignity therapy: a mixed methods study achieving 360° feedback
Source: BMC Palliat Care. 2018 May 10;17:73. doi: 10.1186/s12904-018-0326-0 (PMC5944046; doi:10.1186/s12904-018-0326-0)
Supplement: Supplementary file 1 — Interview guide for HCP focus groups. (DOCX 39 kb) [file 12904_2018_326_MOESM1_ESM.docx]

Interview Guideline Focus Group

Summary

- 2 rounds (Mainz and Wuerzburg), moderation SG, time keeper Mai
- Participants: 5 HCP from different professions per each location, all working in palliative care units) – Attention: recruit 6 participants each in case somebody calls of the interview shortly before
- Aim: to identify (individual-) attitudes and opinions 🡪 DT-acceptance by HCP + expert assessment in order to adapt the interview questions (and feedback questionnaire) both in cultural an linguistically means
- **Research Question:
  How do clinicians working in a palliative care units estimate DT?
  According to experts, which questions of the semi-structured interviews need both linguistic and cultural adaptions?**
- According to experts which questions out of the DT feedback questionnaires need both linguistic and cultural adaptions?
- **[Which reactions does the title evoke (associations, certain feelings)? How is the title’s effect on patients estimated? Are there any suggestions for an alternative naming for this form of intervention in contact with patients (pts.) /** **relatives (R)?]**
- **Realisation:** explorative survey within focus groups – guideline oriented; transcription; qualitative content analysis (developing of categories).
- Focus group duration: 1,5 – 2h.

Equipment

- Informed consent form sheet HCP
- 7 red und 7 green marker pens, 3-5 flipchart-papers
- 6 numbered cards to anonymise the speaker (Flipchart, numbers 1-6)
- 2 audio devices
- 2 notepads (protocol, observations)
- Practical example: Generativity document
- Hand-out Dignity Therapy question protocol (DTQP)
- Hand-out Dignity Therapy Feedback questionnaires pts.+R (FBQ)
- Hand-out task sheet
- Cold drinks and Coffee/Tea
- Chocolate to say ‘Thank you’

| **Basic question** | **Specific requests** | **Realisation, schedule** |
| --- | --- | --- |
| **Beginning: Introduction, Intro** | | |
| Beginning  Own concept, concept DT und pilot study (short introduction)  Introduction Participants: please short, name and function, institution, basic training  Procedure, rules of communication *(no need to put one’s hand up to be allowed to speak, please mention your personal number when beginning to talk, allow others to finish their sentences, beware of balanced* *speaking time-proportions)* | Issues and relevance focus groups, thanks, voluntariness, data protection and privacy statement,  Duration 1,5-2 h | In plenum  10+5 min  *🡪 goal: get to know each other, topical introduction* |
| **1. Block: Brainstorming** | | |
| Basic appeal  Introducing DT by means of a practical example |  | 10 min |
| Brainstorming  “Please let us collect together on the flipchart:  What does spontaneously come to your mind concerning DT?”   - Which benefits / use / positive effects do you see for patients, relatives, colleagues? - Which disadvantages / risks / negative effects could be connected with using DT for pts., R, HCP?   Please make notes about ideas / thoughts / key words on the particular flipcharts, oriented on aspects already mentioned (put themes centered and in large print, draw running branches and lines as well as branch forks)  Conclusion Brainstorming  recourse to the results at the end of focus group | Note:  It does not matter that you build up a strong mind about DT already; firstly we would like to get an overview of all possible aspects, pros and cons that need to be considered (there is no right or wrong). | In plenum  Flipchart, everyone is walking around, holding a pen,  Participants  (SG, Mai take notes)  10+5 min  *🡪 goal: warm-up, determining global acceptance* |
| **2. Block: Revision Question Protocol** | | |
| Group Discussion  I now kindly ask you to thoroughly read the question catalogue.   - How do you think about the question protocol in general?   Let’s go through the question protocol together from top to bottom:   - How could single questions affect patients? Which positive/negative reactions (cognitive/affective) would you expect and why? - Are there any words that particularly catch your eye in a positive/negative way? If so, why? - According to your opinion, which questions do make sense and which do not? - What are the reasons for this estimation? - Do you have any proposed amendments (concrete/linguistically, quantity, question’s length)? | Consider various other perspectives:   - Effects on patients - Cultural dimension - Linguistic dimension/wording - Amount, order and length of questions - [Application by therapists: What could it mean to the therapist to ask these questions?] - [Effects on relatives: How could single questions or their answers affect them?]   Is there anything else you would like to add? Is there anything left to say? | In plenum  Group discussion  Hand-out question protocol (questions numbered)  Audio-recording  20 min  *🡪 goal: expert assessment of interview questions* |
| Summary/ interim conclusion question protocol DT  Asking the plenum while picking up particular important (e.g. especially frequent mentioned) points: „According to your opinion, does this summarize the main points?” | Key words:   - Overall impression question protocol - positive/negative effect particular questions - Relevance particular questions - Estimation usability (amount of questions, length of individual questions, comprehensibility) | In plenum  Audio-recording  5 min |

| **3. Block: Revision feedback questionnaires patients and relatives** | | |
| --- | --- | --- |
| Group work, (alternatively, in case of time running short: together in plenum)  Groups of 2 or 3 (or 3 x FBQ-pts. + 2 x FBQ-R)  (composition preferably heterogeneous between professions)  Discussion und answer to the following questions:   - What is your overall impression of the feedback questionnaire (concerning e.g. auf structure/length, amount and order of questions, scaling? - How could single questions affect patients (in feedback questionnaire pts.) or relatives (in feedback questionnaire R (positive/negative associations/feelings) and why? - Are there any words that particularly catch your eye in a positive/negative way and why? - According to your opinion, which questions are making sense and which do not? - What are the reasons for this estimation? - Do you have any proposed amendments (structure, linguistically)? - Can you think of an aspect that is not mentioned at the moment but needs to be surveyed? |  | Group work (2 groups)  Hand-out Feedback questionnaire pts.+R  Hand-out work sheets  participants  observations  audio-recording  15 min  *🡪 goal: expert assessment of feedback questionnaires* |
| Presentation of results found within the groups  Short, result-oriented – main result, max. 2 min. per group |  | In plenum  Audio-recording  5 min |

| **Bundling findings and conclusion** | | |
| --- | --- | --- |
| Bundling findings  Focus group summary   - Is this summed up correctly?   Reference to results noted on flipchart   - Spontaneous impression DT, estimation of feasibility 🡪 impression after focus group? Collect 2-3 statements of participants   Conclusion   - Is there anything you would like to be remembered concerning the linguistic-cultural adaption and the following implementation of DT? - Do you have any further questions?   Saying thank you for participating in this discussion (small present like chocolate)  Check: Informed consent completed? |  | In plenum  Audio-recording  5+5 min |

| **Impetus for maintenance** |
| --- |
| Is there anything left to say? Addressing participants that do not talk directly while restricting those who talk a lot diplomatically. |
