# Supplementary material for: Feasibility, acceptability and adaption of dignity therapy: a mixed methods study achieving 360° feedback
Source: BMC Palliat Care. 2018 May 10;17:73. doi: 10.1186/s12904-018-0326-0 (PMC5944046; doi:10.1186/s12904-018-0326-0)
Supplement: Supplementary file 2 — Interview guide for patient cognitive interviews. (DOC 80 kb) [file 12904_2018_326_MOESM2_ESM.doc]

Interview Guideline Patients

General comments regarding the interview guideline

This interview guideline is meant to identify patients’ subjective estimations. Basically, open-nded questions are asked as encouragement to start talking, so that interview participants can display their thoughts and opinions freely. Concrete requests are made in cases of uncertainties or to highlight a certain statement. Interviews should take max. 30 minutes, depending, however, on the course (willingness to talk, general condition of the interview-partner) a conversation takes. If new, not already mentioned questions occur during the interview, the interview protocol will be adapted consequently.

Introduction 🡪 Transition after conduction of DT-interviews

Well, Mrs/Mr … now we come to our research interest, to which you have kindly confirmed your support. We have already spoken about the course of the now following conversation in detail. Do you have any last questions about that?

As you already now, we would like to implement Dignity Therapy in Germany. DT has been developed in Canada for patients with challenging health circumstances. Therefore, it is not only necessary to translate the interview questions from English to German but also to check for usability and adapt them linguistically. So this conversation will be about your estimations. I kindly ask you to describe your point of view as concrete and detailed as possible.

Furthermore, I would like to assure you that participating in this conversation and answering to single questions happens voluntarily. You are free to stop this conversation at any point when you feel like not wanting to continue talking or are feeling tired. The audio-recording is confidential and will only be used in encoded form (*informed consent*).

Perception of the title

Frist I would like to ask you to share your perception of this intervention’s title with us.

| Key Questions Title | | |
| --- | --- | --- |
| I would like to kindly ask you to recall that very moment when I suggested to perform Dignity Therapy with you. What thoughts and considerations came to your mind when you heard the title „Dignity Therapy“? | | |
| Content-related Aspects/Checklist | Conversation-sustaining Questions | Requests |
| - Spontaneous impression - Term dignity - Term therapy - Positive/negative associations - Positive/negative feelings - Modification of title yes/no - Title suggestions | Is there anything else that comes to your mind or that you would like to be mentioned?  Is there anything left unsaid? | What associations/feelings does the term dignity evoke?  What associations/feelings does the term therapy evoke?  Should this title be maintained?  What are the reasons for your estimation?  Do you have any request for us regarding modifications? |

Perception of interview questions

The following part will be about estimating the interview protocol. (*Handing out Question Protocol DT with numbers.*) When we conducted the Dignity Therapy interview I already asked you some of these questions. Now I would like to invite you to thoroughly read all of the questions again. Take the time you need.

| Key Questions on Dignity Therapy Question Protocol | | |
| --- | --- | --- |
| I would like to invite you to tell me how you think about the question protocol as a whole? | | |
| Content-related Aspects/Checklist | Conversation-sustaining Questions | Requests |
| - Overall Impression Question Protocol - Estimation usability - Positive/negative effects of individual questions - Relevance of individual questions - Comprehensibility of individual questions - Estimation usability – length individual questions | Is there anything else that comes to your mind or that you would like to be mentioned?  Is there anything left unsaid? | Let us go through the question protocol from top to bottom:  What associations / feelings do the individual questions evoke?  According to your opinion, which questions are making sense and which do not?  What are the reasons for that estimation? (Comprehensibility?)  (How do you think about the length of different questions?) |

| Key Questions actually asked interview questions | | |
| --- | --- | --- |
| Now that we have done the interview together we realise: We did not use every question. When you try to remember which questions we actually talked about: How do you feel about the actually asked interview questions? | | |
| Content-related Aspects/Checklist | Conversation-sustaining Questions | Requests |
| - Overall Impression Question actually asked - Positive/negative effects of individual questions - Relevance of individual questions - Comprehensibility of individual questions | Is there anything else that comes to your mind or that you would like to be mentioned?  Is there anything left unsaid? | Is there a question that is not included within the question protocol but that you liked?  Is there a question that rather should not be asked? |

| Key Questions Words/Wording | | |
| --- | --- | --- |
| Are there any words that particularly draw your attention in a positive / negative way? | | |
| Content-related Aspects/Checklist | Conversation-sustaining Questions | Requests |
| - Positive/negative effects of individual questions - Modification suggestions wording | Is there anything else that comes to your mind or that you would like to be mentioned?  Is there anything left unsaid? | What are the reasons for this?  What associations does this evoke?  What kind of emotions is connected with that?  Is there a word that comes to you mind that you find more suitable? |

| Key Questions proposals for modification | | |
| --- | --- | --- |
| As you know, we would like to adapt the question catalogue to local conditions in both cultural and linguistically means. When you once again take the question protocol into consideration as a whole: What proposals for modification do you have for us? | | |
| Content-related Aspects/Checklist | Conversation-sustaining Questions | Requests |
| - Collecting further questions - Deleting of questions | Is there anything else that comes to your mind or that you would like to be mentioned?  Is there anything left unsaid? | Is there a question you missed in the question catalogue and that needs to be included?  Is there a question in the question catalogue that upset you and should be left out? |

Finishing the interview

We are nearing the end of the interview. Is there anything left that you thing is particularly important and would like to tell or talk about? Do you have further questions?

Conclusion

Thank you very much, that I was allowed to conduct this interview with you. Your information is important for us to be able to prove the question catalogue carefully and adapt it to the local conditions. From my side this interview is finished by now. In case any questions or thoughts might come up later on, we can discuss them during our next meeting where we also will talk about your generativity document which I will have edited until then and will read it aloud for you.

Thank you that you have spent your precious time to participate in this study!
