# Supplementary material for: Feasibility, acceptability and adaption of dignity therapy: a mixed methods study achieving 360° feedback
Source: BMC Palliat Care. 2018 May 10;17:73. doi: 10.1186/s12904-018-0326-0 (PMC5944046; doi:10.1186/s12904-018-0326-0)
Supplement: Supplementary file 3 — DT Patient feedback questionnaire. (DOC 99 kb) [file 12904_2018_326_MOESM3_ESM.doc]

DT Patient Feedback Questionnaire

We would be grateful if you share your feedback and experience of the Dignity Therapy with us. Please answer the following statements:

1. I have found the Dignity Therapy to be helpful to me.

strongly

disagree

disagree

neither agree or disagree

agree

strongly agree

What are your reasons for that opinion?

2. I have found the Dignity Therapy to be satisfactory.

strongly

disagree

disagree

neither agree or disagree

agree

strongly agree

What are your reasons for that opinion?

3. The Dignity Therapy made me feel that my life currently is more meaningful.

strongly

disagree

disagree

neither agree or disagree

agree

strongly agree

What are your reasons for that opinion?

4. The Dignity Therapy has given me a heightened sense of purpose.

strongly

disagree

disagree

neither agree or disagree

agree

strongly agree

What are your reasons for that opinion?

5. The Dignity Therapy has given me a heightened sense of dignity.

strongly

disagree

disagree

neither agree or disagree

agree

strongly agree

What are your reasons for that opinion?

6. The Dignity Therapy has lessened my sense of suffering.

strongly

disagree

disagree

neither agree or disagree

agree

strongly agree

What are your reasons for that opinion?

7. The Dignity Therapy has increased my will to live.

strongly

disagree

disagree

neither agree or disagree

agree

strongly agree

What are your reasons for that opinion?

8. I believe the Dignity Therapy has or will be of help to my family.

strongly

disagree

disagree

neither agree or disagree

agree

strongly agree

What are your reasons for that opinion?

9. I believe my participation in the Dignity Therapy could change the way my family sees or
appreciates me.

strongly

disagree

disagree

neither agree or disagree

agree

strongly agree

What are your reasons for that opinion?

10. I believe my participation in the Dignity Therapy could change the way my health care providers see or appreciate me.

strongly

disagree

disagree

neither agree or disagree

agree

strongly agree

What are your reasons for that opinion?

11. In general, I have been satisfied with my psychosocial care.

strongly

disagree

disagree

neither agree or disagree

agree

strongly agree

What are your reasons for that opinion?

Any other comments regarding your experience of the Dignity Therapy, and how you think it might be improved, would be most appreciated.

Thank you for completing the questionnaire!
