# Supplementary material for: Feasibility, acceptability and adaption of dignity therapy: a mixed methods study achieving 360° feedback
Source: BMC Palliat Care. 2018 May 10;17:73. doi: 10.1186/s12904-018-0326-0 (PMC5944046; doi:10.1186/s12904-018-0326-0)
Supplement: Supplementary file 4 — DT Family feedback questionnaire. (DOC 95 kb) [file 12904_2018_326_MOESM4_ESM.doc]

DT Family Feedback Questionnaire

Initials Relative: ______ Family ID: ______ Patient ID: ______

Gender: male __ female __ Age: ____

# Length of time you have known patient: (ie. indicate number of days, months, years) __________

# Relationship to Patient

Spouce ____ Sibling ____ Child Other relative____

Friend ____ Other (please specify) _____________________

We are interested in your feelings and reactions to the Dignity Therapy intervention that your family member participated in. Please read each of the following statements, and circle the answer that best fits with your opinion.

Did your family member mention the Dignity Therapy Intervention to you? If so, what do you recall them saying?

______________________________________________________________________________________________________________________________________________________________________________________________________________________________________________________

Your thoughts on how this might have helped your family member: Answer each of the following statements, choosing an answer ranging from ‘strongly disagree’ to ‘strongly agree’.

1. I believe Dignity Therapy was helpful to my loved family member.

strongly

disagree

disagree

neither agree or disagree

agree

strongly agree

What are your reasons for that opinion?

2. I believe Dignity Therapy helped to give my family member a heightened sense of purpose or meaning in his life.

strongly

disagree

disagree

neither agree or disagree

agree

strongly agree

What are your reasons for that opinion?

3. I believe Dignity Therapy helped to increase my family member’s sense of dignity

strongly

disagree

disagree

neither agree or disagree

agree

strongly agree

What are your reasons for that opinion?

4. I believe Dignity Therapy helped prepare my family member for death.

strongly

disagree

disagree

neither agree or disagree

agree

strongly agree

What are your reasons for that opinion?

5. I believe Dignity Therapy was as important a component of my family member’s care as any other aspect of their care, including pain management.

strongly

disagree

disagree

neither agree or disagree

agree

strongly agree

What are your reasons for that opinion?

6. I believe Dignity Therapy helped reduce my family member’s suffering.

strongly

disagree

disagree

neither agree or disagree

agree

strongly agree

What are your reasons for that opinion?

7. Do you have any other comments about how you think the Dignity Therapy Intervention helped or affected your family member?

______________________________________________________________________________________________________________________________________________________________________________________________________________________________________________________

Your thoughts on how the Dignity Therapy Intervention might have helped you or your family.

8. Dignity Therapy helps me during my time of grief.

strongly

disagree

disagree

neither agree or disagree

agree

strongly agree

What are your reasons for that opinion?

9. Dignity Therapy will continue to be a source of comfort for my family and me.

strongly

disagree

disagree

neither agree or disagree

agree

strongly agree

What are your reasons for that opinion?

10. I would recommend Dignity Therapy to other patients or family members who are dealing with a terminal illness.

strongly

disagree

disagree

neither agree or disagree

agree

strongly agree

What are your reasons for that opinion?

Can you think of any way we could improve the Dignity Therapy Intervention to make it more helpful?
______________________________________________________________________________________________________________________________________________________________________________________________________________________________________________________

Did your family member experience any negative effects of participating in the Dignity Therapy Intervention?
______________________________________________________________________________________________________________________________________________________________________________________________________________________________________________________

Did you experience any negative effects of participating in the Dignity Therapy Intervention?
______________________________________________________________________________________________________________________________________________________________________________________________________________________________________________________

Do you have any other comments about how you think the **Dignity Therapy Intervention** helped you and/or your family? ______________________________________________________________________________________________________________________________________________________________________________________________________________________________________________________

Thank you for completing the questionnaire!
