# Supplementary material for: Feasibility, acceptability and adaption of dignity therapy: a mixed methods study achieving 360° feedback
Source: BMC Palliat Care. 2018 May 10;17:73. doi: 10.1186/s12904-018-0326-0 (PMC5944046; doi:10.1186/s12904-018-0326-0)
Supplement: Supplementary file 5 — German version of the Dignity Therapy Question Protocol. (DOC 24 kb) [file 12904_2018_326_MOESM5_ESM.doc]

Würdezentrierte Therapie

Erzählen Sie mir ein wenig aus Ihrer Lebensgeschichte; insbesondere über die Zeiten, die Sie am besten in Erinnerung haben oder die für Sie am wichtigsten sind.

Wann haben Sie sich besonders lebendig gefühlt?

Gibt es etwas Besonderes, das Sie Ihrer Familie über sich mitteilen wollen?

Gibt es bestimmte Dinge, die Ihre Familie von Ihnen in Erinnerung behalten sollen?

Was sind die wichtigsten Aufgabenbereiche, die Sie in Ihrem Leben eingenommen haben (Rollen in der Familie, im Beruf, im Sozialleben etc.)?

Warum waren Ihnen diese Aufgaben wichtig und was haben Sie Ihrer Meinung nach darin erreicht?

Was sind Ihre wichtigsten Leistungen, worauf sind Sie besonders stolz?

Gibt es etwas, von dem Sie merken, dass es gegenüber Ihren Lieben noch ausgesprochen werden will?

Oder etwas, das Sie gerne noch einmal sagen möchten?

Was sind Ihre Hoffnungen und Wünsche für die Menschen, die Ihnen am Herzen liegen?

Was haben Sie über das Leben gelernt, das Sie gerne an andere weitergeben möchten?

Welchen Rat oder welche Worte, die Ihre/n … (Tochter, Sohn, Ehemann, Ehefrau, Eltern, anderen Menschen) leiten können, würden Sie gerne weitergeben?

Gibt es konkrete Empfehlungen, die Sie Ihrer Familie mitgeben möchten, um sie für die Zukunft vorzubereiten?

Gibt es speziell für dieses Dokument noch etwas, das Sie hier mit aufnehmen wollen?
